# A High-Precision Machine Learning Algorithm to Classify Left and Right Outflow Tract Ventricular Tachycardia

**DOI:** 10.3389/fphys.2021.641066

**Published:** 2021-02-25

**Authors:** Jianwei Zheng, Guohua Fu, Islam Abudayyeh, Magdi Yacoub, Anthony Chang, William W. Feaster, Louis Ehwerhemuepha, Hesham El-Askary, Xianfeng Du, Bin He, Mingjun Feng, Yibo Yu, Binhao Wang, Jing Liu, Hai Yao, Huimin Chu, Cyril Rakovski

**Affiliations:** ^1^Computational and Data Science, Chapman University, Orange, CA, United States; ^2^Department of Cardiology, Ningbo First Hospital of Zhejiang University, Hangzhou, China; ^3^Department of Cardiology, Loma Linda University, Loma Linda, CA, United States; ^4^Harefield Heart Science Center, Imperial College London, London, United Kingdom; ^5^CHOC Children’s Hospital, Orange, CA, United States; ^6^Department of Environmental Sciences, Faculty of Science, Alexandria University, Alexandria, Egypt; ^7^Zhejiang Cachet Jetboom Medical Devices Co., Ltd., Hangzhou, China

**Keywords:** outflow tract ventricular tachycardia, catheter ablation, electrocardiography, classification, artificial intelligence algorithm

## Abstract

**Introduction:**

Multiple algorithms based on 12-lead ECG measurements have been proposed to identify the right ventricular outflow tract (RVOT) and left ventricular outflow tract (LVOT) locations from which ventricular tachycardia (VT) and frequent premature ventricular complex (PVC) originate. However, a clinical-grade machine learning algorithm that automatically analyzes characteristics of 12-lead ECGs and predicts RVOT or LVOT origins of VT and PVC is not currently available. The effective ablation sites of RVOT and LVOT, confirmed by a successful ablation procedure, provide evidence to create RVOT and LVOT labels for the machine learning model.

**Methods:**

We randomly sampled training, validation, and testing data sets from 420 patients who underwent successful catheter ablation (CA) to treat VT or PVC, containing 340 (81%), 38 (9%), and 42 (10%) patients, respectively. We iteratively trained a machine learning algorithm supplied with 1,600,800 features extracted *via* our proprietary algorithm from 12-lead ECGs of the patients in the training cohort. The area under the curve (AUC) of the receiver operating characteristic curve was calculated from the internal validation data set to choose an optimal discretization cutoff threshold.

**Results:**

The proposed approach attained the following performance: accuracy (ACC) of 97.62 (87.44–99.99), weighted F1-score of 98.46 (90–100), AUC of 98.99 (96.89–100), sensitivity (SE) of 96.97 (82.54–99.89), and specificity (SP) of 100 (62.97–100).

**Conclusions:**

The proposed multistage diagnostic scheme attained clinical-grade precision of prediction for LVOT and RVOT locations of VT origin with fewer applicability restrictions than prior studies.

## Introduction

One population-based study (Dukes et al., 2015) of 1,139 older adults without any heart-failure signs or systolic dysfunction shows that premature ventricular complexes (PVC) and ventricular tachycardia (VT) burden are significantly associated with an increased risk of adjusted decreased left ventricular ejection fraction (odds ratio, 1.13) and increased adjusted risk of incident heart failure (hazard ratio, 1.06) and death (hazard ratio, 1.04). Catheter ablation (CA) is a commonly considered treatment of VT patients with and without structural heart disease when drugs are ineffective or have unacceptable side effects (Cronin et al., 2019). It has a class I indication for treatment of idiopathic outflow tract ventricular tachycardia (OTVT) (Joshi and Wilber, 2005; Latchamsetty et al., 2015). The OTVT stems from the right ventricular outflow tract (RVOT) in 60–80% of the cases and from the left ventricular outflow tract (LVOT) (Bunch and Day, 2006) in the rest of the cases. An accurate prediction of RVOT and LVOT origins of OTVT can optimize the CA strategy, reduce ablation duration, and avoid operative complications. Previous studies (Kamakura et al., 1998; Hachiya et al., 2000; Ito et al., 2003; Joshi and Wilber, 2005; Tanner et al., 2005; Haqqani et al., 2009; Zhang et al., 2009; Betensky et al., 2011; Yoshida et al., 2011, 2014; Cheng et al., 2013, 2018; Nakano et al., 2014; Efimova et al., 2015; He et al., 2018; Xie et al., 2018; Di et al., 2019; Enriquez et al., 2019; Yamada, 2019) propose several criteria or models to estimate RVOT and LVOT origins. However, these results have been limited by sample size, scope of studies, ECG measurement efficiency, and generalizability of the models. In contrast, we develop an optimal multistage scheme that automatically extracts features from standard 12-lead ECGs and incorporates these features into a machine learning model to predict RVOT and LVOT origins of VT or PVC with clinical-grade precision and provides multiprospective analysis for the most important ECG features.

## Materials and Methods

### Study Design

The institutional review board of Ningbo First Hospital of Zhejiang University has approved this retrospective study and granted a waiver of the requirement to obtain informed consent. The study was conducted in accordance with the Declaration of Helsinki.

**FIGURE 1:**
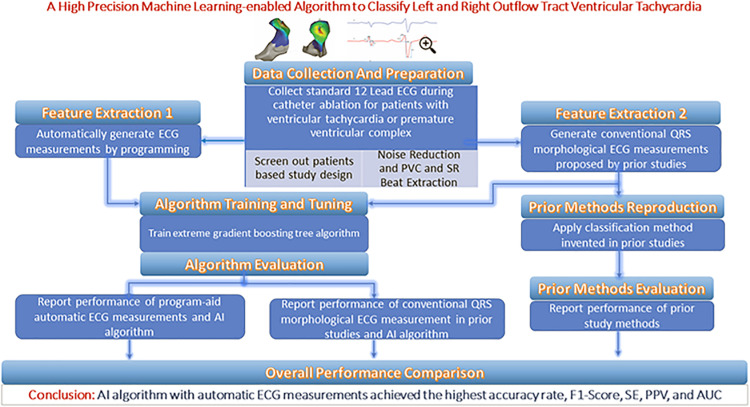
Central illustration.

From each patient’s entire ECG recorder, three cardiac electrophysiologists (EPs) unanimously selected one QRS complex during the sinus rhythm (SR) and one QRS complex during the PVC or VT as the initial input. The features extracted from the two QRS complexes are supplied to an optimal machine learning classification model that provides two possible prediction outputs: RVOT or LVOT. For the purposes of the classification scheme, RVOT is considered a positive outcome and LVOT a negative one. This study employed a training–validation–testing design to correctly assess the performance of the algorithm. This study consists of four phases (shown in Figure 1): (Dukes et al., 2015) a feature extraction phase in which two feature extraction methods are studied and compared—our proprietary automated ECG feature extraction method and a method based on conventional QRS morphological ECG measurements (Cronin et al., 2019) a training phase in which the extreme gradient boosting tree classification model is supplied by the features generated in the feature extraction phase (Joshi and Wilber, 2005) a validation phase aimed at finding important features as optimal model input and deciding the optimal discretization cutoff threshold that was applied in the testing phase; and (Latchamsetty et al., 2015) a testing phase aimed at evaluating, interpreting, and reporting the model performance.

### Patient Selection

We reviewed patients who underwent mapping and ablation for frequent PVC or VT that originated from either LVOT or RVOT at the Ningbo First Hospital of Zhejiang University from March 2007 to September 2019. A PVC or VT burden above 10% of total test duration was required for a study entry. A total of 420 patients with OTVT were included in this study. Origin sites of OTVT were confirmed by a successful CA, which means the frequent PVC and VT did not occur above 5% of the total test duration in the first 6-month follow-up after CA.

### Classification of Anatomic Sites

**FIGURE 2:**
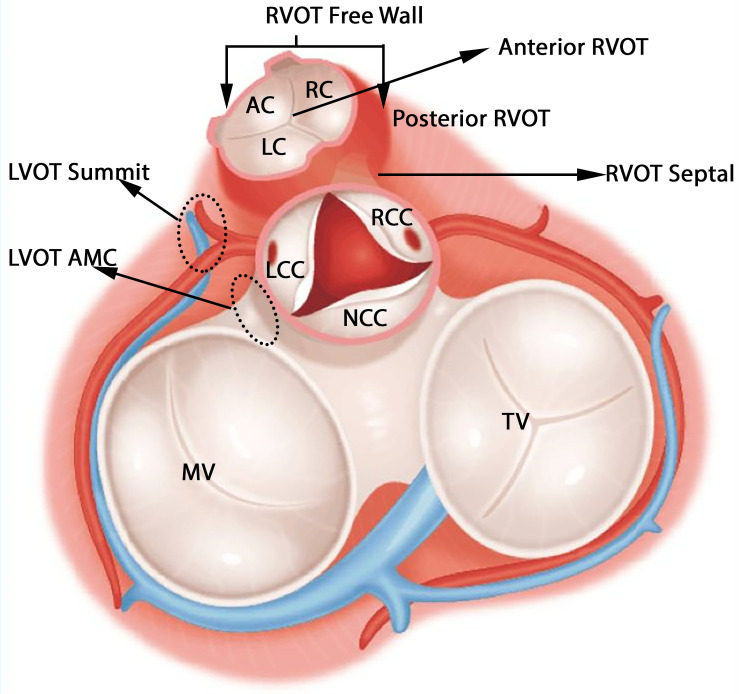
Anatomic structure of LVOT and RVOT. LVOT includes left coronary cusp (LCC), right coronary cusp (RCC), non-coronary cusp (NCC), aortomitral continuity (AMC), and LVOT summit. RVOT includes anterior cusp (AC), left cusp (LC), right cusp (RC), RVOT freewall, and RVOT septal.

The anatomical structure of RVOT and LVOT is depicted in Figure 2, and the demographic data of the anatomic sites are shown in Supplementary Section A and Table 1. This study only focuses on the prediction of RVOT and LVOT rather than the subsites (shown in Figure 2) under RVOT and LVOT. The effective ablation sites of RVOT and LVOT confirmed by ablation provide evidence to create RVOT and LVOT labels for the subsequent machine learning model development.

**TABLE 1:** Summary statistics of demographic data and clinical characteristics of all patients.

	Training cohort			Validation cohort			Testing cohort		
	
	RVOT	LVOT	*P*-value	RVOT	LVOT	*P*-Value	RVOT	LVOT	*P*-Value
Patients, *n* (%)	263 (77)	77 (23)	0.921	30 (79)	8 (21)	0.991	33 (79)	9 (21)	<0.01
Age, year, mean ± sd	46.5 ± 10.6	47.5 ± 11.3	0.731	45.1 ± 13.8	46.9 ± 9.2	0.74	43.8 ± 15.0	45.3 ± 17.1	0.652
Male, *n* (%)	80 (66)	41 (34)	<0.01	6 (46)	7 (54)	<0.01	7 (58)	5 (42)	<0.01
BMI (kg/m^2^), mean ± sd	28.33 ± 3.24	29.28 ± 2.19	<0.01	30.11 ± 3.17	28.37 ± 4.53	<0.01	27.62 ± 4.15	28.37 ± 4.72	<0.01
PVC, n/24 h mean ± sd	, 28,455.5 ± 9,635.8	29,358.5 ± 12,117.4	0.651	30,356.5 ± 18,587.8	276,565 ± 10,997.8	0.531	23,218.5 ± 11,755.6	33,035.6 ± 18,256.3	0.0273
Frequent PVC, *n* (%)	249 (78)	70 (22)	0.683	22 (76)	7 (24)	0.818	28 (85)	4 (44.5)	0.046
Paroxysm VT, *n* (%)	14 (78)	4 (22)	1	1	0	1	2 (6)	4 (44.5)	0.023
Sustained VT, *n* (%)	6 (67)	3 (34)	0.425	1	1	0.398	3 (9)	1 (11)	1
VT cycle length (ms), mean ± sd	410 ± 57	424 ± 102	0.431	426 ± 74	430 ± 88	0.621	438 ± 93	402 ± 147	0.886
Pre-QRS activation time (ms), mean ± sd	29.38 ± 10.26	31.27 ± 8.25	0.334	33.40 ± 5.51	31.65 ± 8.76	0.63	28.64 ± 9.69	33.48 ± 8.46	0.5
Prior CA, n	2	1	0.533	0	0	1	2	0	1
Myocardiopathy,*n*	1	2	0.127	1	0	1	1	1	0.398
Alcoholic cardiomyopathy, *n*	1	0	1	0	0	1	0	0	1
ICD (VT), *n*	1	0	1	0	0	1	1	0	1
Coronary heart disease, *n*	3	1	1	1	0	1	1	1	0.398

*P-values present the probabilities of equal means or proportions of each tested variable. LVOT, left ventricular outflow tract; RVOT, right ventricular outflow tract; BMI, body mass index; LVEF, left ventricular ejection fraction; PVC, premature ventricular complex; VT, ventricular tachycardia; CA, catheter ablation.*

### Mapping and Ablation Procedure

**FIGURE 3:**
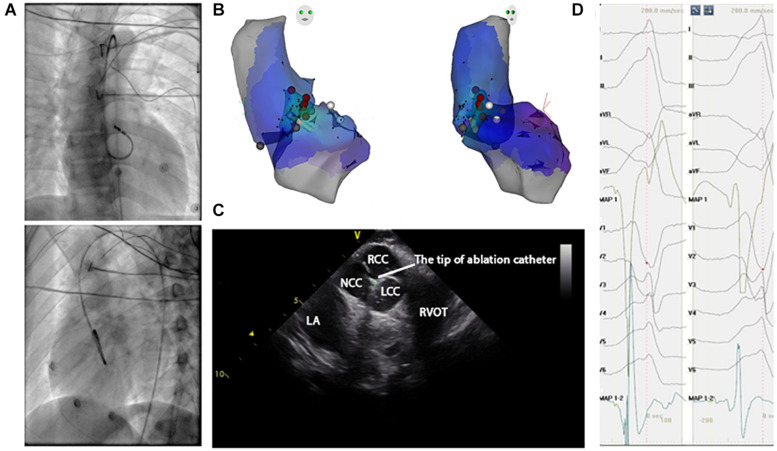
Activation map and fluoroscopic map for VA originating from commissure of aortic sinus of valsalva in LVOT. **(A)** Right and left anterior oblique fluoroscopic views show an ablation catheter in the LVOT. Ablation in the LVOT (LCC–RCC commissure) eliminated the PVC within 3 s. **(B)** The 3-D anatomic representation of the RV endocardium, LV endocardium, and venous system with the ablation catheter positioned at the anterior interventricular vein. **(C)** The green circle indicates the tip of the ablation catheter in LCC–RCC commisure. **(D)** The earliest bipolar and unipolar activation time (–30 ms) are shown.

Anti-arrhythmic drugs were stopped for at least five half-lives before the inception of the ablation procedure. A 4.0-mm 7F irrigated ablation catheter (Navistar; Biosense Webster, Diamond Bar, CA, United States) was initially placed in the RVOT for mapping. Both fluoroscopy and electroanatomic mapping systems (CARTO, Biosense Webster, Diamond Bar, CA, United States or NavX Velocity, St. Jude Medical, St. Paul, MN, United States) were used to localize the anatomic position of the ablation catheter within the outflow tract. The intracardiac echo was used to identify specific anatomical structures, such as cusps and papillary muscles. For example, Figure 3 presents the fluoroscopy, 3-D mapping, intracardiac echocardiography, and activation mapping for a case with the origin site in commissure of aortic sinus of valsalva LVOT. Using point-by-point mapping, anatomic aggregated maps were created. Activation mapping was performed in all patients during VT and PVC. Pace mapping was also performed with the lowest pacing output (2–20 mA) and pulse width (0.5–10 ms) to capture the ventricular myocardium at the site of the earliest activation. If suitable ablation sites for the RVOT were not located or ablation failed to abolish the arrhythmia, extended mapping to the LVOT site was deployed *via* a retrograde aortic approach. After target sites were located, radiofrequency energy was delivered up to a maximum power of 35 W and a maximum electrode-tissue interface temperature of 43°C. If the VT or PVC disappeared or the frequency of arrhythmias diminished after the first 30 s of ablation, the energy was delivered continuously from 60 to 180 s. Ablation success was defined as the absence of spontaneous or induced VT or PVC at 30 min after the last energy delivery and confirmed by continuous cardiac telemetry in the subsequent 24 h of inpatient care.

### The Procedure to Assess the Catheter Ablation Outcomes

In the subsequent 24 h of inpatient care after the ablation procedure, every patient received continuous ECG monitoring. After discharge, the patients underwent a follow-up 2 weeks after the ablation and then every month at the cardiology clinic. A 12-lead surface ECG test was obtained on each clinic visit, and 24-h Holter monitoring was also prescribed at 3 and 6 months after the ablation.

### ECG Measurement Protocol

#### Noise Reduction and QRS Sample Selection

With chest and limb leads placed carefully in a standard position, the 12-lead surface ECGs were collected by the EP workmate system (EP-WorkMate^TM^ System, Abbott, Saint Paul, MI, United States) at a sampling rate of 2,000 Hz before the ablation procedure. The noise sources impacting the ECG database were power line interference, baseline wandering, and random noise. Wavelet transform yields better time–frequency localization results than windowed Fourier transform and naturally has an advantage in noise reduction applications (Abi-Abdallah et al., 2006). Thus, the wavelets technique was used to remove the noise components mentioned above. The coif5 Wavelets (Lahmiri, 2014) and Stein’s Unbiased Risk Estimator (SURE)-based (Stein, 1981; David and Johnstone, 1995) threshold were implemented by MATLAB to carry out the noise reduction steps. To get a full understanding of the techniques and schemes that were adopted in this work, please refer to the code availability section. After noise components were removed, three cardiac EPs unanimously selected one QRS complex during the SR and one QRS complex during the PVC or VT to classify RVOT and LVOT.

#### Automated ECG Feature Extraction Method

**FIGURE 4:**
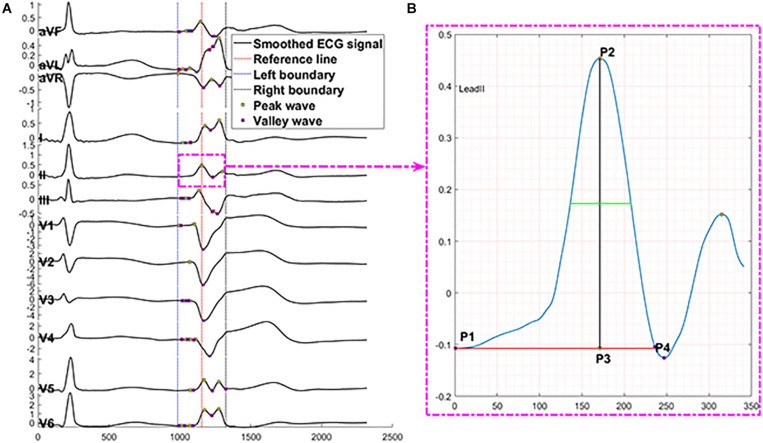
Description of automated ECG feature extraction method. The proposed feature extraction method automatically finds peaks presented by P# and valleys presented by V# in panel **(A)** through 430 data points of one SR beat in 12 leads. Panel **(B)** presents the numerical measurements that capture essential information of a peak, including location = sample points at P3, prominence = distance from P2 to P3, distance from peak or valley location to left prominence boundary = distance from P1 to P3, distance from peak or valley location to right prominence boundary = distance from P3 to P4, width at half of the prominence = the length of green line, distance from left prominence boundary to right prominence boundary = distance from P1 to P4, amplitude = distance from P2 to zero baseline, contour height = prominence – amplitude. *X*-axis presents sampling data points, and *Y*-axis presents voltage.

We applied the following measurements and transformation protocol to automatically extract ECG morphological features and supply them to the machine learning model. We used the R-wave peak points of PVC and SR heartbeat in lead V_6_ as reference lines because they are easy to identify in most conditions. At the first step, for one SR heartbeat, 215 data points (0.11 s) before and after the reference line were truncated, and 335 data points (0.17 s) before and after the reference line were cut for one PVC. The above lengths of 430 and 670 were the means of QRS complex duration plus four times the standard deviation of that for SR beat and PVC. They should cover 99.99% of the QRS complexes in any data due to the normality of the QRS duration distribution and the empirical rule. The mean and standard deviation of QRS duration were computed from the samples in this study; the maximum length of QRS complex for SR beat is 405 data points, and the maximum for PVC is 607 data points. Second, for every lead, we selected the first peak/valley (local maximum or minimum) closest to the reference line (shown in Figure 4A) defined in the first step. Third, the three peaks or valleys before the first peak/valley identified in the second step and the four peaks or valleys after the first peak/valley were selected from all peaks and valleys of SR heartbeat and PVC separately. Thus, in every lead, eight peaks and valleys were extracted to represent the SR heartbeat and PVC basic features. The zero-padding method was applied for the cases that did not have eight peaks and valleys around the reference line. The total number of peaks and valleys, eight, is equal to the means of the number of peaks and valleys in all leads plus four times the standard deviation of that for SR beat and PVC, respectively. This automated feature extraction method was verified manually to make sure it captured essential QRS morphological characteristics.

The numerical measurements (shown in Figure 4B) of each peak and valley include location, prominence, the distance from peak or valley location to left prominence boundary, the distance from peak or valley location to right prominence boundary, width at half of the prominence, the distance from left prominence boundary to right prominence boundary, amplitude, contour height, and a logic variable to present peak or trough. The prominence of a peak or a valley measures how much the peak or valley stood out due to its intrinsic height and location relative to neighbor peaks or valleys. Thus, the prominence of a peak was defined as the vertical distance between the peak point and its lowest contour line. The measurement of valleys adopted the same method with peaks.

After the above eight numerical measurements of eight peaks or valleys for both SR beat and PVC at every lead were collected, we generated a feature matrix with the size of 192 (2 beats × 12 leads × 8 peaks or valleys) by 8 (the number of numerical measurements). We transformed the feature matrix using ratios of features in the rows and columns of the matrix to create a new level of features that can reveal vital details of the ECG morphology. Finally, 1,600,800 features were automatically obtained, and their definitions can be found in Supplementary Section B.2. The estimated 95% CI of each numerical measurement in the feature matrix is documented in Supplementary Section B.2 and Supplementary Table 5.

#### Conventional QRS Morphological Feature Extraction

Even though we intended to develop an automated ECG measurement system that is favored by the machine learning algorithm, the conventional QRS morphological ECG measurement method, such as metrics of Q-, R-, and S-waves; segments among them; and the ratios among segments, is studied and compared in this work. The conventional QRS morphological ECG measurement protocol is defined below. SR and VT ECG morphology were measured on the same 12-lead ECG by a customized MATLAB program. During the clinical arrhythmia, the following measurements (presented in Supplementary Section B.3 and Figure 1) were obtained from both one SR beat and one PVC: (Dukes et al., 2015) amplitude of Q-, R-, and S-waves (Cronin et al., 2019) duration of Q-, R-, and S-waves as well as QRS complex; and (Joshi and Wilber, 2005) R/S amplitude ratio (Kamakura et al., 1998; Ito et al., 2003), transitional zone (Hachiya et al., 2000; Tanner et al., 2005), V_2_ transition ratio (Betensky et al., 2011), transitional zone index (Yoshida et al., 2011; Di et al., 2019), R-wave deflection interval (Cheng et al., 2013), V_2_S/V_3_R index (Yoshida et al., 2014), R-wave duration index (Ouyang et al., 2002), and R/S amplitude index (Ouyang et al., 2002). The T-P segment was considered one of the isoelectric baselines to measure R- and S-wave amplitudes. The QRS duration was measured from the site of the earliest initial deflection from the isoelectric line to the time of the latest activation. The R-wave length was calculated from the site of the earliest initial deflection from the isoelectric line to the time at which the R-wave intersected the isoelectric line. For all cases, QRS measurements were performed on an isolated PVC representative of the clinical VT before the induction of sustained VT and compared with the SR QRS complex. All measurements above were used to compare our approach against methods from 12 prior studies (Kamakura et al., 1998; Zhang et al., 2009; Betensky et al., 2011; Yoshida et al., 2011, 2014; Cheng et al., 2013, 2018; Nakano et al., 2014; Efimova et al., 2015; He et al., 2018; Xie et al., 2018; Di et al., 2019).

In addition to the above conventional ECG measurements, we developed the following protocol to generate features to supply to the machine learning model. Amplitudes of Q-, R-, and S-waves based on the voltage at the onset of Q-wave, the offset of S-wave, the Q-wave, and the S-wave were also input variables in the machine learning model. To give the same length input to the machine learning model, we set the measures of Q-, R-, and S-waves for these waves’ missing cases to zeros, such as QS morphology in the V_1_ lead and RS morphology in the V_5_ or V_6_ lead. As we implemented the automated feature extraction method, we also transformed the measurements mentioned above into new variables and put them into the machine learning model. The total number of features generated by this method is 155,784, and the entire definition of features can be found in Supplementary Section B.3. The 95% CI of each numerical measurement are listed in Supplementary Section B.3 and Supplementary Table 7.

### Statistical Analysis

For the continuous variables of age and ECG measurements, we calculated the mean and standard deviation. For all count variables, total sample size, number of males, number of subjects with frequent PVC, sustained VT, and sublocations under RVOT or LVOT, we calculated frequency counts and percentages. One-sample test for proportions, two-sample *t* test, two-sample test for proportions, and Fisher’s exact test were adopted to test the difference of the sample numbers, average ages, genders, and the number of frequent PVC or sustained VT between RVOT and LVOT groups. The Cramer Von Mises, Anderson–Darling, and Shapiro–Wilks tests did not reject the data normality hypothesis, and a two-sample *t* test was used to test for equal means of continuous variables between RVOT and LVOT. Statistical optimization of the gradient boosting tree model was done through iterative training using the extreme gradient booster (XGBoost) package. The following performance measures were formally analyzed, including the area under the curve (AUC) of the receiver operating characteristic (ROC) curve, accuracy (ACC), sensitivity (SE), specificity (SP), and F1-score. A two-sided 95% CI summarizes the sample variability in the estimates. The CI for the AUC was estimated using the Sun and Su optimization of the Delong method implemented in the pROC package. In contrast, CIs for F_1_-score, SE, and SP were obtained by the bootstrap method with 20,000 replications. All analyses were done by R version 3.5.3.

## Results

We analyzed data from 420 patients who underwent CA of OTVT at the Ningbo First Hospital of Zhejiang University from March 2007 to September 2019. After the CA procedure, two (0.5%) patients developed slight ecchymosis. A total of five (1.2%) patients were excluded from this study because of frequent PVC or VT recurrence in the first 6-month follow-up.

Patient demographic and clinical characteristics data for the RVOT and LVOT groups are shown in Table 1. We compare the distributions of these background characteristics in the RVOT and LVOT groups and list the associated *p*-values in the table. The RVOT cohort consists of 20.95% left cusp, 17.62% posterior septal, 14.29% anterior septal, 10% anterior cusp, 7.86% free wall, and 7.14% right cusp. The LVOT cohort consists of 10.71% left coronary cusp, 5.71% aortomitral continuity, 2.62% left coronary cusp and right coronary cusp ommisure, 1.67% right coronary cusp, and 1.43% summit (shown in Supplementary Section A and Table 1).

The patients were assigned to training, validation, and testing cohorts, consisting of 340 (81%), 38 (9%), and 42 (10%) patients, respectively, using random proportional allocation (demographic summary shown in Table 1). For a fair comparison, the machine learning model was supplied with different features from two feature extraction methods. The performance was assessed using the same training, validation, and testing cohorts.

**FIGURE 5:**
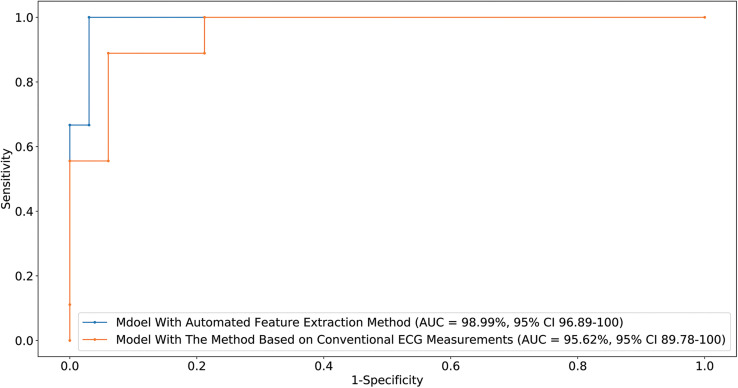
Receiver-operating characteristic curve generated by the optimal machine learning model supplied with two feature extraction methods. The CI for the AUC was estimated using the Sun and Su optimization of the Delong method. Sensitivity and specificity of RVOT prediction are indicated for different thresholds.

**FIGURE 6:**
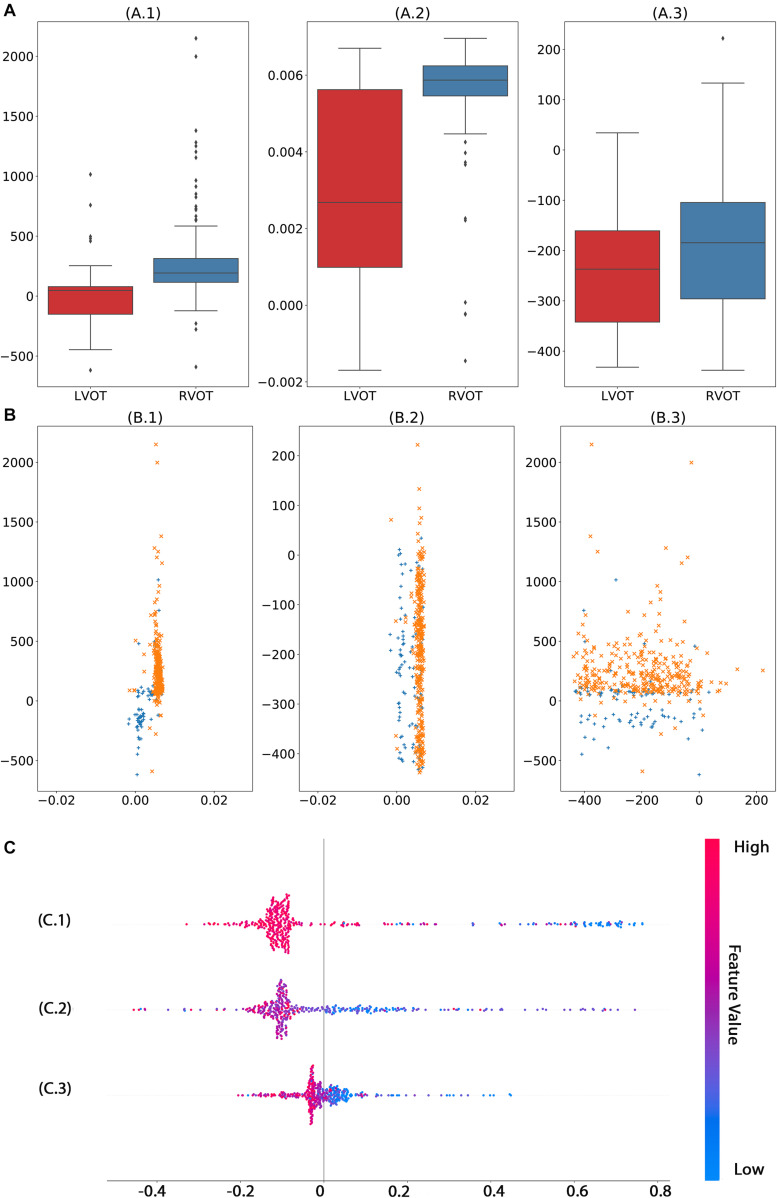
Analysis of top three significant ECG measurements found by machine learning model with automated feature extraction method. The univariate analysis **(A)** shows that features 1 **(A.1)** and 2 **(A.2)** have significant capability to separate RVOT and LVOT. The bivariate analysis **(B)** indicates the classification ability of one–one interaction of the top 3 significant features. In the multivariate analysis **(C)**, the smaller feature 1 **(C.1)**, feature 1 **(C.2)**, and feature 3 **(C.3)** generate a higher probability of LVOT, but the magnitude of influence varies across features. The color in **panel (C)** represents the feature value (red high, blue low).

We used 1,600,800 automatically generated ECG features as machine learning model input. The proposed approach achieved an ACC of 97.62 (87.44–99.99); F_1_-score of 98.46 (90–100); prediction of RVOT origins with SE of 96.97 (82.54–99.89); and SP of 100 (62.97–100) (shown in Table 2), respectively; and AUC of 98.99 (96.89–100) (presented in Figure 5). Among the 1,600,800 initial automatically generated ECG features, we found a total of 1,352 critically important features with non-zero Shapley additive explanations (SHAP) values (Lundberg and Lee, 2017), showing the importance of their contributions to RVOT and LVOT prediction. The detailed interpretation of SHAP value is introduced in Supplementary Section C.1. We chose and analyzed the top three important features (shown in Figure 6) that have significant classification capability: (Dukes et al., 2015) the ratio between the location of the 5th peak or valley at the SR beat V_1_ lead and the right boundary of the 5th peak or valley at the V_1_ lead of PVC, Cronin et al. (2019) the ratio between the prominence of the 5th peak or valley at the V1 lead of PVC and the prominence of the 5th peak or valley at the V3 lead of PVC, and (Joshi and Wilber, 2005) the difference between the distance of the 5th peak or valley to the left boundary at the V1 lead of PVC and the distance of the 5th peak or valley to the left boundary at the V_1_ lead of the SR beat.

**TABLE 2:** Classification performance comparison with 95% CI.

	AUC	SE	SP	F_1_-Score	ACC
Automated ECG feature extraction	98.99% (96.89–100)	96.97% (82.54–99.89)	100% (62.97–100)	98.46% (90–100)	97.62% (87.44–99.99)
Conventional QRS morphological feature extraction	95.62% (89.78–100)	93.94% (78.64–98.99)	88.89% (50.86–99.45)	95.38% (86.62–98.86)	92.86% (80.35–98.85)
Cardiologists	NA	97.86%	81.72%	96.39%	94.29%

*F_1_-score = 2 × Precision × recall / (precision + recall); SE, sensitivity; SP, specificity; ACC, accuracy; CI, confidence interval.*

Training the machine learning model using 155,784 features extracted from conventional QRS morphological ECG measurements, the proposed method attained an ACC of 92.86 (80.35–98.85), F_1_-score of 95.38 (86.62–98.86), prediction of RVOT origins with SE of 93.94 (78.64–98.99) and SP of 88.89 (50.86–99.45) (shown in Table 2), and AUC of 95.62 (89.78–100) (presented in Figure 5). Among the initial 155,784 features, we found a total of 1,003 critically important features with non-zero SHAP values (Lundberg and Lee, 2017), showing the importance of their contributions to RVOT and LVOT prediction. The top three important features (shown in Supplementary Section C.1 and Figure 2) that show significant classification capability are (Dukes et al., 2015) the ratio between R-wave amplitude based on the zero isoelectric baselines at lead III PVC and the R-wave amplitude based on the offset of S-wave at V_1_ lead PVC, Cronin et al. (2019) the ratio between the R-wave amplitude based on R-wave onset at V_2_ lead SR beat and the *R*-wave amplitude based on zero isoelectric baseline at V_3_ lead PVC, and (Joshi and Wilber, 2005) the ratio between the R-wave amplitude based on the zero isoelectric baseline at aVL lead SR beat and the R-wave amplitude based on S-wave offset at V_1_ lead PVC. The statistical summary of conventional QRS morphological measurements for leads V_1_ to V_6_ is listed in Supplementary Section A and Table 2.

Finally, the average performance of eight cardiologists who determined RVOT and LVOT using the same ECG samples in this study is presented in Table 2. The classification confusion matrix for these three methods shows correct and incorrect frequency counts in Supplementary Section A and Table 3. Furthermore, we compared our approach against related methods from 12 prior studies (Kamakura et al., 1998; Zhang et al., 2009; Betensky et al., 2011; Yoshida et al., 2011, 2014; Cheng et al., 2013, 2018; Nakano et al., 2014; Efimova et al., 2015; He et al., 2018; Xie et al., 2018; Di et al., 2019). ACC, F_1_-score, SE, SP, positive predictive value, negative predictive value, and AUC were used to compare performances and are shown in Table 3.

**TABLE 3:** Comparison with prior studies to localize the origins of outflow tract arrhythmia.

Author	Patients	ECG criteria/algorithm	Reported performance in the article	Performance using the database in this study
This Study	420	1,600,800 ECG criteria and extreme gradient boosting tree model	SE 96.97% SP 100% PPV 100% NPV 90% AUC 98.99% ACC 97.62% F_1_-Score 98.46%	
Kamakura et al. (1998)	40	The R/S transition (first precordial lead with R/S ration >1) in Lead V_3_ to predict LVOT	SE 80% PPV 40% SP 82.86% NPV 96.67%	SE 30.1% PPV 18.92% SP 63.3% NPV 73.4%
Zhang et al. (2009)	65	(a) Transitional zone ≥ V_4_ predicts RVOT origin	SE 94.87% PPV 100%	SE 60.86% PPV 72.47%
		(b) R-wave duration index <0.5 and R/S wave amplitude index <0.3 in V1/V2 predicts RVOT origin	SE 94.87% PPV 100%	SE 80.24% PPV 73.52%
Betensky et al. (2011)	61	(a) V_2_ transition ratio (defined as percentage R wave during VT divided by percentage R wave in SR) ≥ 0.6 predicts LVOT origin	SE 95% SP 100% PPV 100% NPV 95% ACC 91%	SE 78.49% SP 89.6% PPV 68.22% NPV 93.61% ACC 87.14%
		(b) PVC precordial transition later than SR transition predicts RVOT origin	SE 19% SP 100%	SE 23% SP81%
Yoshida et al. (2011)	207	V_2_S/V_3_R index ≤ 1.5 predicts LVOT origin	SE 89% SP 94% PPV 84% NPV 96%	SE 69.89% SP 85.63% PPV 58.04% NPV 90.9%
Cheng et al. (2013)	94	(a) R/S transition at lead V_1_/V_2_ predicts LVOT origin	SE 52.4% SP 92.1% PPV 72.6% NPV 85.3% ACC 84.2%	SE 76.34% SP 91.43% PPV 71.72% NPV 93.15% ACC 88.09%
		(b) R/S transition at lead V_3_ predicts RVOT origin	SE 39% SP 35.2% PPV 74.2% NPV 29.4% ACC 46.3%	SE 33.33% SP 48.93% PPV 15.66% NPV 72.07% ACC 45.48%
		(c) R/S transition at lead V_4_ or later predicts RVOT origin	SE 59.3% SP 93.1% PPV 94.6% NPV 46.7% ACC 68.3%	SE 43.01% SP 52.6% PPV 20.51% NPV 76.44% ACC 50.47%
Yoshida et al. (2014)	112	TZ index = TZ score of OTVT minus TZ score of a sinus beat	To aortic sinus cusp SE 88% SP 82% AUC 0.9	SE 76.05% SP 52.59%
Nakano et al. (2014)	63	(a) R > S concordance in synthesized right-sided chest leads (Syn-V_3_R, Syn-V_4_R, Syn-V_5_R) predicts an LVOT origin	SE 100% SP 100%	Could not be reproduced by standard 12-lead ECG
		(b) R/S index (>0.3): A ratio of R-wave amplitude to S-wave amplitude in leads V_1_ or V_2_ predicts an LVOT origin	SE 90% SP 98%	SE 53.12% SP 46.05%
Efimova et al. (2015)	105	A QRS-RVA (right ventricular apex) interval ≥ 0.49 ms predicts an LVOT origin. The QRS-RVA interval was measured from the onset of the QRS complex to the distal RVA signal.	SE 98%, SP 94.6%, PPR 94.1%, NPR 98.1%, ACC 96.1%	Could not be reproduced by standard 12-lead ECG
Cheng et al. (2018)	94	R-wave deflection interval in lead V_3_ > 80 ms and R-wave amplitude index in lead V_1_	SE 100% SP 83% PPV 85.7% NPV 100% ACC 91.7%	SE 59.14% SP 58.1% PPV 28.64% NPV 83.33% ACC 58.33%
He et al. (2018)	488	*Y* = −1.15*(TZ) − 0.494*(V_2_S/V_3_R)	SE 90% SP 87% AUC 0.88%	SE 78.39% SP 67.23% AUC 0.79%
Xie et al. (2018)	75	R-wave amplitude ≥ 0.1 mV to predict LVOT	SE 75% SP 98% PPV 92.3% NPV 93% AUC 0.85%	SE 67.74% SP 58.1% PPV 31.5% NPV 86.36%
Di et al. (2019)	184	V_1_-V_3_ transition index to predict RVOT	SE 93% SP 86% AUC 0.931 ACC 95%	SE 70.33% SP 67.74% ACC 69.76%

*The first column presents the first author name and the reference number in the main text; TZ, transition zone; SE, sensitivity; SP, specificity; PPV, positive predictive value; NPV, native predictive value; ACC, accuracy; AUC, area under curve.*

## Discussion

We designed and implemented a high-accuracy algorithm for LVOT and RVOT origins of OTVT classification, using 1,600,800 ECG measurements automatically extracted from 12-lead ECGs using our proprietary method. The prediction accuracy comparison among our method combined with the XGBoost classifier, a conventional QRS feature extraction method combined with XGBoost, and the performance of human experts (shown in Table 2) shows that the machine learning model with the automated ECG feature extraction method was uniformly superior. We used DeLong’s test (DeLong et al., 1988) to demonstrate that the automated ECG feature extraction method had a significantly higher AUC compared with that attained by the conventional QRS morphological feature extraction approach with a *P*-value = 0.035. The comparison of our approach against methods from 12 prior studies (Kamakura et al., 1998; Zhang et al., 2009; Betensky et al., 2011; Yoshida et al., 2011, 2014; Cheng et al., 2013, 2018; Nakano et al., 2014; Efimova et al., 2015; He et al., 2018; Xie et al., 2018; Di et al., 2019) shows that our algorithm achieved the highest performance scores (shown in Table 3). Additionally, we evaluated the general classification capability of each criterion proposed by previous studies using the database in this study. Not surprisingly, we observed significant differences between previously reported performances and the reproduced results of these methods because most of the prior studies used the univariate analysis to make predictions (shown in Table 3).

The excellent performance of our machine learning algorithm demands an enormous volume of data and features. It is an extremely time- and cost-consuming task to generate such amount of features by the conventional ECG QRS morphological measurements introduced in prior studies because these measurements are manually obtained. Thus, we did not make any assumptions about ECG criteria before training the machine learning algorithm and intended to exhaust all possible relationships among morphological measures of Q-, R-, and S-waves as well as the entire QRS complex. We designed and implemented an automated ECG feature extraction method that can generate 1,600,800 ECG signal characteristics. Not only did these features contain a considerable amount of the classical statistics from 12 prior studies (Kamakura et al., 1998; Zhang et al., 2009; Betensky et al., 2011; Yoshida et al., 2011, 2014; Cheng et al., 2013, 2018; Nakano et al., 2014; Efimova et al., 2015; He et al., 2018; Xie et al., 2018; Di et al., 2019), but they also captured morphological measures not considered by previous studies, such as rsR’ waves and rsr’s’ waves. However, one may be concerned that such a feature extraction method will include the P- and T-wave within SR beats and retrograde P-waves within PVC. The machine learning model captures and analyzes a large amount of information from every beat but filters out all unimportant features based on their classification accuracy contribution. As we can see from the top three important features (shown in Figure 6) selected by the machine learning model, none of the features that presented waves mentioned above played a role in the prediction. The important morphological features of the Rsr’ and rsr’s waves may be caused by noise and lead placement of the 12-lead ECG electrodes because the 12-lead ECG electrodes are frequently misplaced due to the mapping patches used during the ablation procedure. In this study, we avoid such a problem because chest and limb leads were placed carefully in a standard position when the 12-lead surface ECGs were collected before the procedure.

Moreover, before the machine learning model is safely applied in practice, an unambiguous interoperation is necessary for cardiologists to gear this advanced tool, such as explaining what crucial criteria are and why they play vital roles. For instance, the machine learning model shows that the smaller the magnitude of the first important feature (shown in Figure 6C.1), the higher the possibility of LVOT origin of OTVT. The first important feature is the ratio of the location of the 5th peak or valley at the V1 lead SR beat and the right boundary of the 5th peak or valley at the V_1_ lead of PVC. In our feature extraction system, the 5th peak or valley at the V_1_ lead of PVC is an S-wave in most cases. The key ECG lead in the initial site prediction of VT origin is the V_1_ lead because it is located nearly orthogonal to the septal plane and, thus, is the best lead to resolve initial right- vs. left-sided activation. When the V_1_ lead has a positive QRS (R > s), the VT is considered to have the right bundle branch block (RBBB) configuration. Conversely, net negative QRS (*r* < S) defines a left bundle branch block (LBBB) configuration (Haqqani and Marchlinski, 2019). The top three important features (shown in Figure 6) were exactly measured activation time, RBBB, and LBBB configuration. Therefore, such interpretation makes the machine learning decision process not a black box anymore.

Last but not least, the machine learning model proposed in this study can be immediately and effortlessly deployed to EP labs. The pretrained model, source code, and data are available online and found in the “Data Availability Statement” section. The model inputs are only two QRS complexes, one for PVC and one for SR beat, and they can be easily acquired from 12-lead standard ECG. The analysis of one patient’s data takes less than a second provided every step of measurement and computation is automatically done by the model and the preprocessing approach. The precise prediction of origins can significantly reduce CA duration and reduce the risk of complications.

### Study Limitations

Because the data set did not produce enough well-labeled data to feed a machine learning model, the algorithm currently only predicts LVOT and RVOT rather than subsites of them. For instance, the origin of PVC is sometimes in the middle of septal RVOT/LVOT. The presence of expertly labeled data for three categories, RVOT, LVOT, and septal, will allow the machine learning model to predict the origins with higher accuracy. Although this study includes patients with comprehensive anatomy sites under RVOT and LVOT, the performance of the method could improve in the presence of more cases of RCC and summit under LVOT. Moreover, some conditions, such as cardiomyopathies, reentrant VT coronary heart disease, and prior structural and congenital abnormalities, are underrepresented or absent from the study. Thus, the algorithm potentially has a limitation if applied in such scenarios.

## Conclusion

Considering the performance of prediction, the capacity of extracting vital information from 12-lead ECG and the robustness of application, our results provide the promising and reliable decision support to guide a successful CA treatment of ventricular arrhythmia by machine learning technology.

## Data Availability Statement

The datasets presented in this study can be found in online repositories. The names of the repository/repositories and accession number(s) can be found below: https://doi.org/10.6084/m9.figshare.c.4668086.v2.

## Author Contributions

JZ, GF, XD, BH, HC, and CR processed the data for analysis. JZ, HC, GF, XD, IA, and CR performed the statistical analysis. All authors contributed to the study design, data interpretation, and writing of the report.

## Conflict of Interest

From the Department of Cardiology, Ningbo First Hospital of Zhejiang University. HC has served as a consultant for Biosense Webster, Boston Scientific, and Abbott. HY was employed by the company Zhejiang Cachet Jetboom Medical Devices Co., Ltd. The remaining authors declare that the research was conducted in the absence of any commercial or financial relationships that could be construed as a potential conflict of interest.
