# Supplementary material for: A High-Precision Machine Learning Algorithm to Classify Left and Right Outflow Tract Ventricular Tachycardia
Source: Front Physiol. 2021 Feb 25;12:641066. doi: 10.3389/fphys.2021.641066 (PMC7947246; doi:10.3389/fphys.2021.641066)
Supplement: Supplementary file 1 [file Table_1.DOCX]

**A High Precision Machine Learning-enabled Algorithm to Classify Left and Right Outflow Tract Ventricular Tachycardia**

Jianwei Zheng, PhD1*†, Guohua Fu, MD2†, Islam Abudayyeh, MD, MPH3, Sir Magdi Yacoub4, Anthony Chang, MD, MPH5, William W. Feaster, MD5, Louis Ehwerhemuepha, PhD5, Hesham El-Askary, PhD1,6, Xianfeng Du, MD2, Bin He, MD2, Mingjun Feng, MD2, Yibo Yu, MD2, Binhao Wang, MD2, Jing Liu, MD2, Hai Yao7, Huimin Chu, MD2*, Cyril Rakovski PhD1

1: Chapman University

2: Department of Cardiology, Ningbo First Hospital of Zhejiang University

3: Department of Cardiology, Loma Linda University Health

4: Harefield Heart Science Center, Imperial College London

5: CHOC Children's Hospital

6: Department of Environmental Sciences, Science, Alexandria University,

7: Zhejiang Cachet Jetboom Medical Devices CO.LTD

**Corresponding Author:**

Huimin Chu

Address: 59 Liuting St, Haishu, Ningbo, Zhejiang, China, 315016

Phone: +86 574 8708 5588

Fax: +86 574 87291583

Email: mark.chuhuimin@gmail.com

**Supplementary Material**

**Section A.**

**Table 1: [Summary Statistics of All Anatomic Sites.]**^[[1]](#footnote-1)^

| Left/Right | Sub Locations | Number (%) |
| --- | --- | --- |
| RVOT | LC | 88 (20.95) |
|  | Posterior Septal | 74 (17.62) |
|  | Anterior Septal | 60 (14.29) |
|  | AC | 42 (10) |
|  | Free wall | 33 (7.86) |
|  | RC | 30 (7.14) |
| LVOT | LCC | 45 (10.71) |
|  | AMC | 24 (5.71) |
|  | LCC-RCC Ommisure | 11 (2.62) |
|  | RCC | 7 (1.67) |
|  | Summit | 6 (1.43) |

**Table 2: [Electrocardiographic Measurements and Successful Ablation Sites in All Patients.**] ^[[2]](#footnote-2)^

|  | RVOT | LVOT | P-Value |
| --- | --- | --- | --- |
| Lead V1 |  | | |
| R-wave amplitude (mV) SR | 0.36±0.33 | 0.32±0.28 | 0.267 |
| S-wave amplitude (mV) SR | 0.98±0.55 | 1.04±0.55 | 0.377 |
| R-wave duration (ms) SR | 28.25±16.08 | 25.63±27 | 0.052 |
| QRS duration (ms) SR | 58.26±15.02 | 61.06±13.42 | 0.175 |
| R/S ratio SR | 0.97±0.27 | 0.55±0.47 | 0.481 |
| R-wave duration ratio SR | 1.44±0.34 | 1.34±0.26 | 0.683 |
| R-wave amplitude (mV) PVC | 0.32±0.27 | 0.85±0.59 | <0.001 |
| S-wave amplitude (mV) PVC | 1.81±0.77 | 0.55±0.63 | <0.001 |
| R-wave duration (ms) PVC | 19.55±3.09 | 22.33±4.03 | <0.001 |
| QRS duration (ms) PVC | 60.09±12.02 | 61.11±11.04 | <0.001 |
| R/S ratio PVC | 0.28±0.19 | 0.39±0.24 | <0.001 |
| R-wave duration ratio PVC | 1.41±0.13 | 1.44±0.19 | 0.435 |
| Leave V_2_ |  | | |
| R-wave amplitude (mV) SR | 0.77±0.48 | 0.75±0.41 | 0.6 |
| S-wave amplitude (mV) SR | 1.58±0.78 | 1.74±0.85 | 0.078 |
| R-wave duration (ms) SR | 29.82±4.16 | 32.85±6.17 | 0.083 |
| QRS duration (ms) SR | 66.29±18.02 | 67.06±19.01 | 0.928 |
| R/S ratio SR | 0.47±0.27 | 0.42±0.25 | 0.511 |
| R-wave duration ratio SR | 1.27±0.19 | 1.28±0.21 | 0.356 |
| R-wave amplitude (mV) PVC | 0.59±0.4 | 1.74±0.97 | <0.001 |
| S-wave amplitude (mV) PVC | 3.1±1.33 | 1.08±1.07 | <0.001 |
| R-wave duration (ms) PVC | 29.55±5.09 | 34.63±6.09 | <0.001 |
| QRS duration (ms) PVC | 69.1±22.02 | 69.12±17.04 | <0.001 |
| R/S ratio PVC | 0.33±0.23 | 0.48±0.19 | <0.001 |
| R-wave duration ratio PVC | 1.34±0.24 | 1.32±0.13 | 0.003 |
| Lead V_3_ |  | | |
| R-wave amplitude (mV) SR | 1.18±0.64 | 1.21±0.66 | 0.659 |
| S-wave amplitude (mV) SR | 0.92±0.62 | 1.24±0.69 | <0.001 |
| R-wave duration (ms) SR | 30.83±12.16 | 29.87±14.17 | 0.099 |
| QRS duration (ms) SR | 60.06±25.01 | 61.06±24.01 | 0.869 |
| R/S ratio SR | 1.36±0.12 | 1.57±0.21 | 0.063 |
| R-wave duration ratio SR | 1.51±0.36 | 1.48±0.33 | 0.221 |
| R-wave amplitude (mV) PVC | 1.02±0.56 | 2.77±1.14 | <0.001 |
| S-wave amplitude (mV) PVC | 1.4±1.22 | 0.35±0.64 | <0.001 |
| R-wave duration (ms) PVC | 30.59±15.09 | 31.65±14.09 | <0.001 |
| QRS duration (ms) PVC | 65.12±27.03 | 70.14±24.03 | <0.001 |
| R/S ratio PVC | 1.46±0.55 | 1.52±0.67 | <0.001 |
| R-wave duration ratio PVC | 1.56±0.28 | 1.55±0.31 | 0.005 |
| Lead V_4_ |  | | |
| R-wave amplitude (mV) SR | 1.76±0.71 | 1.92±0.79 | 0.058 |
| S-wave amplitude (mV) SR | 0.5±0.41 | 0.71±0.48 | <0.001 |
| R-wave duration (ms) SR | 32.84±8.16 | 34.87±6.89 | 0.111 |
| QRS duration (ms) SR | 76±25.01 | 75.06±22.01 | 0.2 |
| R/S ratio SR | 1.56±0.64 | 1.76±.51 | 0.335 |
| R-wave duration ratio SR | 1.75±0.21 | 1.74±0.25 | 0.015 |
| R-wave amplitude (mV) PVC | 1.53±0.71 | 3.3±1 | <0.001 |
| S-wave amplitude (mV) PVC | 0.23±0.46 | 0.05±0.1 | <0.001 |
| R-wave duration (ms) PVC | 33.64±8.1 | 30.68±6.09 | 0.001 |
| QRS duration (ms) PVC | 76.15±29.03 | 74.16±26.03 | 0.037 |
| R/S ratio PVC | 2.72±1.11 | 2.92±1.24 | 0.306 |
| R-wave duration ratio PVC | 2.59±0.64 | 2.57±0.46 | 0.668 |
| Lead V_5_ |  | | |
| R-wave amplitude (mV) SR | 1.76±0.67 | 1.96±0.77 | 0.014 |
| S-wave amplitude (mV) SR | 0.26±0.25 | 0.35±0.3 | 0.004 |
| R-wave duration (ms) SR | 30.84±6.16 | 31.87±5.96 | 0.086 |
| QRS duration (ms) SR | 75.36±27.01 | 77.06±25.01 | 0.84 |
| R/S ratio SR | 3.16±1.35 | 4.02±1.94 | 0.659 |
| R-wave duration ratio SR | 2.69±0.72 | 2.74±0.63 | 0.163 |
| R-wave amplitude (mV) PVC | 1.84±0.69 | 3±0.93 | <0.001 |
| S-wave amplitude (mV) PVC | 0.03±0.16 | 0.02±0.05 | 0.673 |
| R-wave duration (ms) PVC | 37.67±10.1 | 40.68±13.09 | 0.184 |
| QRS duration (ms) PVC | 79.17±18.03 | 81.16±21.03 | 0.329 |
| R/S ratio PVC | 9.04±3.04 | 9.77±3.97 | 0.578 |
| R-wave duration ratio PVC | 3.12±0.92 | 4.29±0.66 | 0.1 |
| Lead V_6_ |  | | |
| R-wave amplitude (mV) SR | 1.5±0.55 | 1.62±0.65 | 0.073 |
| S-wave amplitude (mV) SR | 0.15±0.27 | 0.18±0.2 | 0.308 |
| R-wave duration (ms) SR | 40.84±22.16 | 38.88±27.16 | 0.067 |
| QRS duration (ms) SR | 75.06±26.01 | 77.07±28.31 | 0.17 |
| R/S ratio SR | 11.08±4.43 | 11.63±4.97 | 0.699 |
| R-wave duration ratio SR | 13.7±3.28 | 13.88±3.33 | 0.645 |
| R-wave amplitude (mV) PVC | 1.83±0.59 | 2.39±0.75 | <0.001 |
| S-wave amplitude (mV) PVC | 0.02±0.13 | 0.02±0.05 | 0.898 |
| R-wave duration (ms) PVC | 45.67±22.12 | 48.68±26.09 | 0.424 |
| QRS duration (ms) PVC | 78.17±19.03 | 80.16±22.07 | 0.178 |
| R/S ratio PVC | 23.88±9.15 | 27.45±8.09 | 0.231 |
| R-wave duration ratio PVC | 4.08±0.75 | 4.27±0.65 | 0.024 |

**Table 3: [Classification Confusion Matrix of Optimal Artificial Intelligence Model and Cardiologists.]** ^[[3]](#footnote-3)^

| True Conditions |  | Predicted Conditions | | |
| --- | --- | --- | --- | --- |
|  |  |  | LVOT | RVOT |
|  | Auto Features | LVOT | 9 | 0 |
|  |  | RVOT | 1 | 32 |
|  | QRS Features | LVOT | 8 | 1 |
|  |  | RVOT | 2 | 31 |
|  | Cardiologists | LVOT | 76 | 17 |
|  |  | RVOT | 7 | 320 |

**Section B.**

**B.1 The full version of the ECG measurement protocol.**

The 12-lead ECGs during the whole ablation procedure were collected at a sampling rate of 2,000 Hz. Standard 12-lead ECG electrode placement was used. In this work, the coif5 Wavelets and SURE-based threshold were implemented by MATLAB for noise reduction.

We applied the following measurements and transformation protocol to automatically extract ECG morphological features and feed them into the machine learning model. We used the R-wave peak time points of PVC and SR heartbeat in the lead V_6_ as reference lines since they are easy to be identified in most conditions. At the first step, for one SR heartbeat, 215 data points (0.11 seconds) before the reference line and 215 data points after the reference line were truncated, and 335 data points (0.17 seconds) before the reference line and 335 data points after the reference line were cut for one PVC. The above lengths of 430 and 670 were the means of QRS complex duration plus four times the standard deviation of that for SR beat and PVC, respectively, and should cover 99.99% of the QRS complexes in any data due to the normality of the distribution of the QRS duration and an application of the empirical rule. The mean and standard deviation of QRS duration were computed from the samples in this study, the maximum length of QRS complex for SR beat is 405 data points, and that for PVC is 607 data points. Secondly, for every lead we selected the first peak/valley (local maximum or minimum) closest to the reference line defined in the first step. Thirdly, the three peaks or valleys before the first peak/valley that was identified in the second step, and the four peaks or valleys after the first peak/valley were selected from all peaks and valleys of SR heartbeat and PVC, separately. Thus, in every lead, eight peaks and valleys were extracted to represent the basic features of the SR heartbeat and PVC . For the cases that do not have eight peaks and valleys around the reference line, the zero-padding method was applied. The total number of peaks and valleys, eight is equal to the means of the number of peaks and valleys in all leads plus four times the standard deviation of that for SR beat and PVC, respectively. This automated feature extraction method was verified manually to make sure it can capture essential QRS morphological characteristics.

The numerical measurements of each peak and valley included location, prominence, the distance from peak or valley location to left prominence boundary, the distance from peak or valley location to right prominence boundary, width at half of the prominence, the distance from left prominence boundary to right prominence boundary, amplitude, contour height, and a logic variable to present peak or trough. The prominence of a peak or a valley measures how much the peak or valley stood out due to its intrinsic height and location relative to neighbor peaks or valleys. Thus, the prominence of a peak was defined as the vertical distance between the peak point and its lowest contour line. The measurement of valleys adopted the same method with peaks.

After the above eight numerical measurements of eight peaks or valleys for both SR beat and PVC at every lead were collected, we generated a feature matrix with the size of 192 (2 beats * 12 leads * 8 peaks or valleys) by 8 (the number of numerical measurements). We transformed the feature matrix to create enormous new criteria that can reveal the vital ECG morphological information, such as the ratios of the row-wise feature matrix and the ratios of the column-wise feature matrix. Finally, 1,600,800 features were automatically obtained, and the manifest of them can be found in section B.2. The estimated margin error within 95% CI of each numerical measurement in the feature matrix was documented in section B.2 Table 5.

Even though we intended to develop an automated ECG measurement system that is favored by the machine learning algorithm, the conventional QRS morphological ECG measurement method, such as metrics of Q-, R-, and S-waves, segments among them, and the ratios among segments, was studied and compared in this work. The conventional QRS morphological ECG measurements protocol was defined as below. SR and VT ECG morphology were measured on the same 12-lead ECG by customized MATLAB program with precision 1/2000 second. During the clinical arrhythmia, the following measurements (presented in section C.4 Figure 1) were obtained from both one SR beat and one PVC: 1) amplitude of Q-, R-, and S-waves; 2) duration of Q-, R-, and S-waves as well as QRS complex; 3) R/S amplitude ratio ^12, 13^, transitional zone ^14, 15^, V_2_ transition ratio ^18^, transitional zone index ^19, 27^, the R-wave deflection interval ^20^, the V_2_S/V_3_R index ^21^, R-wave duration index^30^(30) and R/S amplitude index^30^. The T-P segment was considered one of the isoelectric baselines for the measurement of R- and S-waves amplitudes. The QRS duration was measured from the site of the earliest initial deflection from the isoelectric line to the time of the latest activation. The R-wave length was measured from the site of the earliest initial deflection from the isoelectric line to the time at which the R-wave intersected the isoelectric line. For all cases, QRS measurements were performed on isolated PVCs representative of the clinical VT before the induction of sustained VT and compared with the SR QRS complex. All measurements above were used to compare our approach against methods from 12 prior studies ^12, 17-27^.

In addition to the above conventional ECG measurements, we developed the following protocol to generate features to feed the machine learning model. Amplitudes of Q-, R- and S-waves based on the voltage at the onset of Q-wave, the offset of S-wave, the Q-wave, and the S-wave were also input variables for the machine learning model. To give the same length input to the machine learning model, we set the zero value to measures of Q-, R- and S-waves for these waves missing cases, such as QS morphology in V_1_ lead, and RS morphology in V_5_ or V_6_ lead. As we implemented for the automated feature extraction method, we also transformed the measurements mentioned above to new variables and put them into the machine learning model. The total number of features generated by this method is 155,784, and the entire definition of features can be found in section C.3. The estimated margin errors within 95% CI of each numerical measurement were listed in section B.3 Table 7.

**B.2 The Manifest of 1,600,800 Automatically Extracted ECG Features**

In this work, we picked eight peaks and valleys (local maximum and minimum) from SR beat and PVC beat, respectively, the 4^th^ peak or trough closest to R-wave peak point in V_6_ lead as the reference line. Therefore, all peaks and troughs in each lead align with the reference line defined above. The measurements of each peak and valley included the location, prominence, distance from peak or valley location to left prominence boundary, distance from peak or valley location to right prominence boundary, width at half of the prominence, distance from left prominence boundary to right prominence boundary, amplitude, and contour height. The above eight variables for 12 lead ECGs both SR beat and PVC were pile up together, generating a feature matrix with the size of 192 by 8 (shown in **Table 4**).

**Table 4: [Feature Matrix Generated by Automated ECG Feature Extraction Method.]**

| Row number |  | Variable 1 | … | Variable 8 |
| --- | --- | --- | --- | --- |
| 1 | Lead aVF, SR beat, the first peak or valley |  |  |  |
| … | … |  |  |  |
| 8 | Lead aVF, SR beat, the 8^th^ peak or valley |  |  |  |
| 9 | Lead aVF, PVC, the first peak or valley |  |  |  |
| … | … |  |  |  |
| 16 | Lead aVF, PVC, the 8^th^ peak or valley |  |  |  |
| … | … |  |  |  |
| 192 | Lead V_6,_ PVC, the 8^th^ peak or valley |  |  |  |

**Table 5: [Estimated Margin Error of Measurements Generated by Automated ECG Feature Extraction Method, Within 95% Confidence Interval.]**

| **Measurement Names** | **Mean ± Estimated Margin Error** |
| --- | --- |
| Prominence | 0.18 **±** 2.4*10^-3^ mV |
| Distance from peak or valley location to left prominence boundary | 0.29 **±** 19.5*10^-5^ second |
| Distance from peak or valley location to right prominence boundary | 0.31 **±** 22.2*10^-5^ second |
| Width at half of the prominence | 0.012 **±** 9.6*10^-5^ second |
| Contour height | 0.33 **±** 4.2*10^-3^ mV |
| Distance from left prominence boundary to right prominence boundary, | 0.028 **±** 22.9*10^-5^ second |
| Amplitude | 0.15 **±** 3.8*10^-3^ mV |

1. ECG measurements within a single lead include:
   1. Location, prominence, distance from peak or valley location to left prominence boundary, distance from peak or valley location to right prominence boundary, width at half of the prominence, distance from left prominence boundary to right prominence boundary, amplitude, contour height, and a logic variable to present peak or trough., totally 9 (the number of variables) * 8 (the number of peaks or valley for each lead) * 2 (the number of PVC and SR beats) *12 (the number of leads) =1,728.
   2. The ratios of the difference (deviation) among peaks and valleys in 8 variables (location, prominence, distance from peak or valley location to left prominence boundary, distance from peak or valley location to right prominence boundary, width at half of the prominence, distance from left prominence boundary to right prominence boundary, amplitude, contour height) for PVC or SR beat, respectively. For each lead, the total number of differences among peaks and valleys in 8 variables is equal to 28 (chosen 2 variables from 8 variables) *8 (the number of variables) for PVC or SR beat. For each lead, the total number of ratios of the differences among peaks and valleys in 8 variables for PVC or SR beat is equal to 378 (chosen 2 differences from 28 differences) * 8 (the number of variables). Thus, for 12 leads, the total number of ratios of the difference among peaks and valleys in 8 variables is equal to 378 * 8 (the number of variables) * 2 (the number of PVC and SR beats) *12 (the number of leads) = 72,576.
2. ECG measurements among 12 leads include:
3. The ratios of the row-wise feature matrix. The total number of ratios is equal to 18,336 (chosen 2 rows from 192 rows) * 8 (the number of variables) = 146,688.
4. The differences of variables row-wise feature matrix. The total number of differences is equal to 18,336 (chosen 2 rows from 192 rows) * 8 (the number of variables) = 146,688.
5. The ratios of the time differences of peaks or troughs vs. the difference calculated in b). The total number of ratios is equal to 18,336 (chosen 2 rows from 192 rows) * 7 (the number of variables) = 128,352.
6. The differences of the column-wise feature matrix. The total number of ratios is equal to 28 (chosen 2 variables from 8 variables) * 192 (the number of rows in feature matrix) = 5,376.
7. The ratios of each item in the feature matrix vs. the left items expect the rations that already computed in part a), b), c), and d). The total number is equal to 1,600,800.

**B.3 The Manifest of 155,784 ECG Measurements (Features) Based on Conventional QRS Morphology Measurements.**

To feed the machine learning model with the same length of variables, we took the alternatives for these Q-wave, R-wave, or S-wave missing from certain leads. For example, Q-wave missing from lead V_1_, we used three consecutive points to present Q-wave onset, Q-wave, and Q-wave offset before R-wave onset. Thus, the duration of Q-wave is very close to zero, and the amplitude is set to zero. The same operation was adapted for R- and S-waves. Every QRS complex has five time points, K, Q, R, S, and J, presenting Q-wave onset, Q-wave, R-wave, S-wave, and S-wave offset, respectively.

1. ECG measurements within a single lead include:

Locations at K, Q, R, S, and J, amplitudes at K, Q, R, S, and J based on zero line, time durations from K to Q, from K to R, from K to S, K to J, from Q to R, from Q to J, from R to S, from R to J, and from S to J, amplitudes at Q, R, S and J based on the voltage value at K, amplitudes at K, Q, R, and S based on the voltage value at J. The total number of variables is equal to 23 * 2(SR beat and PVC) * 12 (the number of leads) = 672.

All variables above excluding the variables of sample points at K, Q, R, S, and J for both SR beat and PVC for every lead were pile up together, generating a feature matrix (demonstrated in **Table 6**) with the size of 12 (the number of leads) by 46 (the number of variables).

**Table 6: [Feature Matrix Generated by Conventional QRS Morphology Measurements.]**

|  | Variable 1 for SR beat | … | Variable 23 for SR beat | Variable 1 for PVC | … | Variable 23 for PVC |
| --- | --- | --- | --- | --- | --- | --- |
| Lead aVF |  |  |  |  |  |  |
| … |  |  |  |  |  |  |
| Lead V_6_ |  |  |  |  |  |  |

**Table 7: [Estimated Margin Error of Measurements Generated by Conventional QRS Morphology Measurements Method, Within 95% Confidence Interval.]**

| **Measurement Names** | **Mean ± Estimated Margin Error** |
| --- | --- |
| Distance from K to Q, SR beat | 0.01 ± 22*10^-5^ second |
| Distance from K to R, SR beat | 0.04 ± 34*10^-5^ second |
| Distance from K to S, SR beat | 0.07 ± 49*10^-5^ second |
| Distance from K to J, SR beat | 0.1 ± 60*10^-5^ second |
| Distance from Q to R, SR beat | 0.03 ± 27*10^-5^ second |
| Distance from Q to S, SR beat | 0.06 ± 43*10^-5^ second |
| Distance from Q to J , SR beat | 0.09 ± 59*10^-5^ second |
| Distance from R to S, SR beat | 0.03 ± 30*10^-5^ second |
| Distance from R to J, SR beat | 0.06 ± 54*10^-5^ second |
| Distance from S to J , SR beat | 0.03 ± 51*10^-5^ second |
| Amplitudes at Q based on the voltage value at K, SR beat | 0.18 ± 9*10^-3^ mV |
| Amplitudes at R based on the voltage value at K, SR beat | 3.86 ± 114*10^-3^ mV |
| Amplitudes at S based on the voltage value at K, SR beat | 2.2 ± 82*10^-3^ mV |
| Amplitudes at J based on the voltage value at K, SR beat | 0.059 ± 16*10^-3^ mV |
| Amplitudes at K based on the voltage value at J, SR beat | 0.059 ± 16*10^-3^ mV |
| Amplitudes at Q based on the voltage value at J, SR beat | 0.115 ± 17*10^-3^ mV |
| Amplitudes at R based on the voltage value at J, SR beat | 3.92 ± 121*10^-3^ mV |
| Amplitudes at S based on the voltage value at J, SR beat | 2.14 ± 86*10^-3^ mV |
| Amplitudes at K based on zero line, SR beat | 0.089 ± 8*10^-3^ mV |
| Amplitudes at Q based on zero line, SR beat | 0.265 ± 11*10^-3^ mV |
| Amplitudes at R based on zero line, SR beat | 3.77 ± 114*10^-3^ mV |
| Amplitudes at S based on zero line, SR beat | 2.29 ± 85*10^-3^ mV |
| Amplitudes at J based on zero line, SR beat | 0.149 ± 15*10^-3^ mV |
| Distance from K to Q, PVC | 0.004 ± 25*10^-5^ second |
| Distance from K to R, PVC | 0.083 ± 103*10^-5^ second |
| Distance from K to S, PVC | 0.142 ± 114*10^-5^ second |
| Distance from K to J, PVC | 0.165 ± 88*10^-5^ second |
| Distance from Q to R, PVC | 0.078 ± 105*10^-5^ second |
| Distance from Q to S, PVC | 0.138 ± 118*10^-5^ second |
| Distance from Q to J , PVC | 0.161 ± 92*10^-5^ second |
| Distance from R to S, PVC | 0.059 ± 56*10^-5^ second |
| Distance from R to J, PVC | 0.082 ± 93*10^-5^ second |
| Distance from S to J , PVC | 0.023 ± 81*10^-5^ second |
| Amplitudes at Q based on the voltage value at K, PVC | 0.048 ± 6*10^-3^ mV |
| Amplitudes at R based on the voltage value at K, PVC | 5.47 ± 164*10^-3^ mV |
| Amplitudes at S based on the voltage value at K, PVC | 3.6 ± 115*10^-3^ mV |
| Amplitudes at J based on the voltage value at K, PVC | 0.733 ± 68*10^-3^ mV |
| Amplitudes at K based on the voltage value at J, PVC | 0.733 ± 68*10^-3^ mV |
| Amplitudes at Q based on the voltage value at J, PVC | 0.685 ± 68*10^-3^ mV |
| Amplitudes at R based on the voltage value at J, PVC | 6.2 ± 212*10^-3^ mV |
| Amplitudes at S based on the voltage value at J, PVC | 2.86 ± 145*10^-3^ mV |
| Amplitudes at K based on zero line, PVC | 0.216 ± 19*10^-3^ mV |
| Amplitudes at Q based on zero line, PVC | 0.264 ± 19*10^-3^ mV |
| Amplitudes at R based on zero line, PVC | 5.25 ± 163*10^-3^ mV |
| Amplitudes at S based on zero line, PVC | 3.81 ± 122*10^-3^ mV |
| Amplitudes at J based on zero line, PVC | 0.95 ± 66*10^-3^ mV |

1. ECG measurements among 12 leads include:
2. The ratios of column-wise of feature matrix, for PVC and SR beats. The total number of these ratios is equal to 1035 (chosen 2 columns from 46 columns) * 12 (the number of rows in feature matrix) = 12,420.
3. The ratios of row-wise of feature matrix, for PVC and SR beats. The total number of ratios is equal to 66 (chosen 2 rows from 12 rows) * 46 (the number of columns) = 3036.
4. The difference of row-wise of feature matrix, for PVC and SR beats. The total number of ratios is equal to 66 (chosen 2 rows from 12 rows) * 46 (the number of columns) = 3036.
5. The ratios of each item in the feature matrix vs. the left elements, excluding the rations that already computed in section a), b), and c). The total number is equal to 136,620.

**Figure 1: [Conventional Electrocardiographic Measurements.** Leads V_1_ to V_3_ of normal sinus heartbeat followed by a premature contraction complex representative of the clinical outflow tract ventricular tachycardia. Measurements are defined as following: RA1 = SR R-wave amplitude (mV); RD1 = SR R-wave duration (ms); SD1 = SR S-wave duration (ms); SA1 = SR S-wave amplitude (mV); RA2 = PVC R-wave amplitude (mV); RD2 = PVC R-wave duration (ms); SD2 = PVC S-wave duration (ms); SA2 = PVC S-wave amplitude (mV); SR onset of R-wave = the start point of RD1; SR offset of R-wave = the end point of RD1; SR onset of S-wave = the start point of SD1; SR offset of S-wave = the end point of SD1]


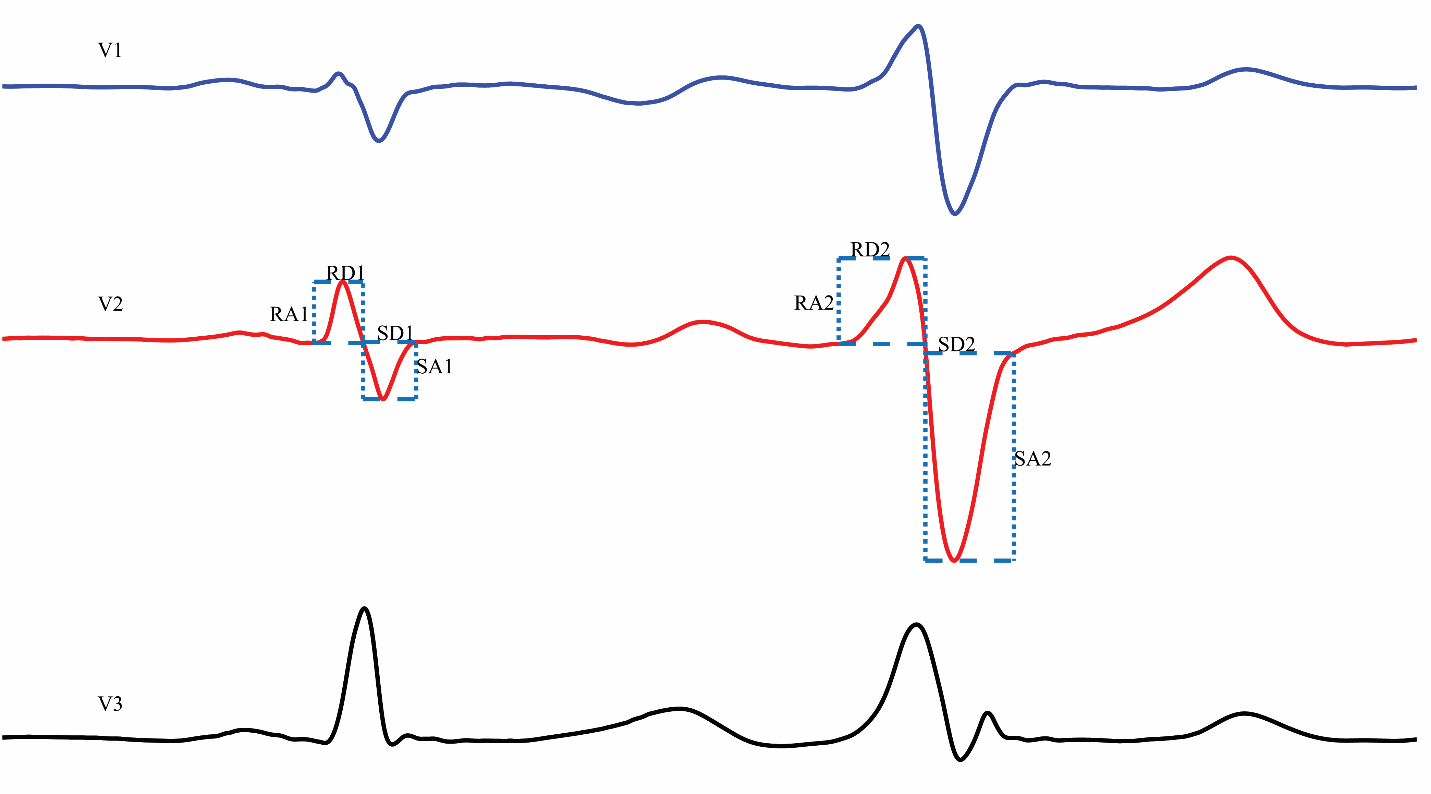


**Section C**

**C.1 Features Interpretation by SHAP Values.**

One of the severe challenges for the artificial intelligence algorithm, especially for deep learning, neural network model, and gradient boosting algorithm, is the explanation of features definition and their influence on the model outcome. In this study, we adapted a novel measure, SHAP value, to illustrate the feature importance. SHAP (Shapley additive explanations) is a game-theoretic approach to explain the output of any machine learning model. It connects optimal credit allocation with local explanations using the classical Shapley values from game theory and their related extensions. The collective SHAP values can show how much each feature contributes, either positively or negatively, to the target variable. It looks like the variable importance plot, but it can show a positive or negative relationship for each variable with the target. Each sample gets its own set of SHAP values, so that dramatically increases the transparency. One can explain why a case receives its prediction and the contributions of the predictors. Traditional variable importance algorithms only show the results across the entire population but not on each case. The local interpretability enables us to pinpoint and contrast the impacts of the factors.

**Figure 2: [The Analysis of Top 3 Significant ECG Measurements Found by Machine Learning Model with Conventional QRS Morphological Feature Extraction Method.** The univariate analysis (A) shows that no single feature can separate RVOT and LOVT linearly. The bivariate analysis (B) shows that precise separation contour does exist too. In the multivariate analysis (C), the higher feature 1 (C.1) and feature 2 (C.2) will generate a higher probability of LVOT, and feature 3 (C.3) plays the opposite. The color in (C) represents the feature value (red high, blue low). The definition of features was introduced in the Result section.]


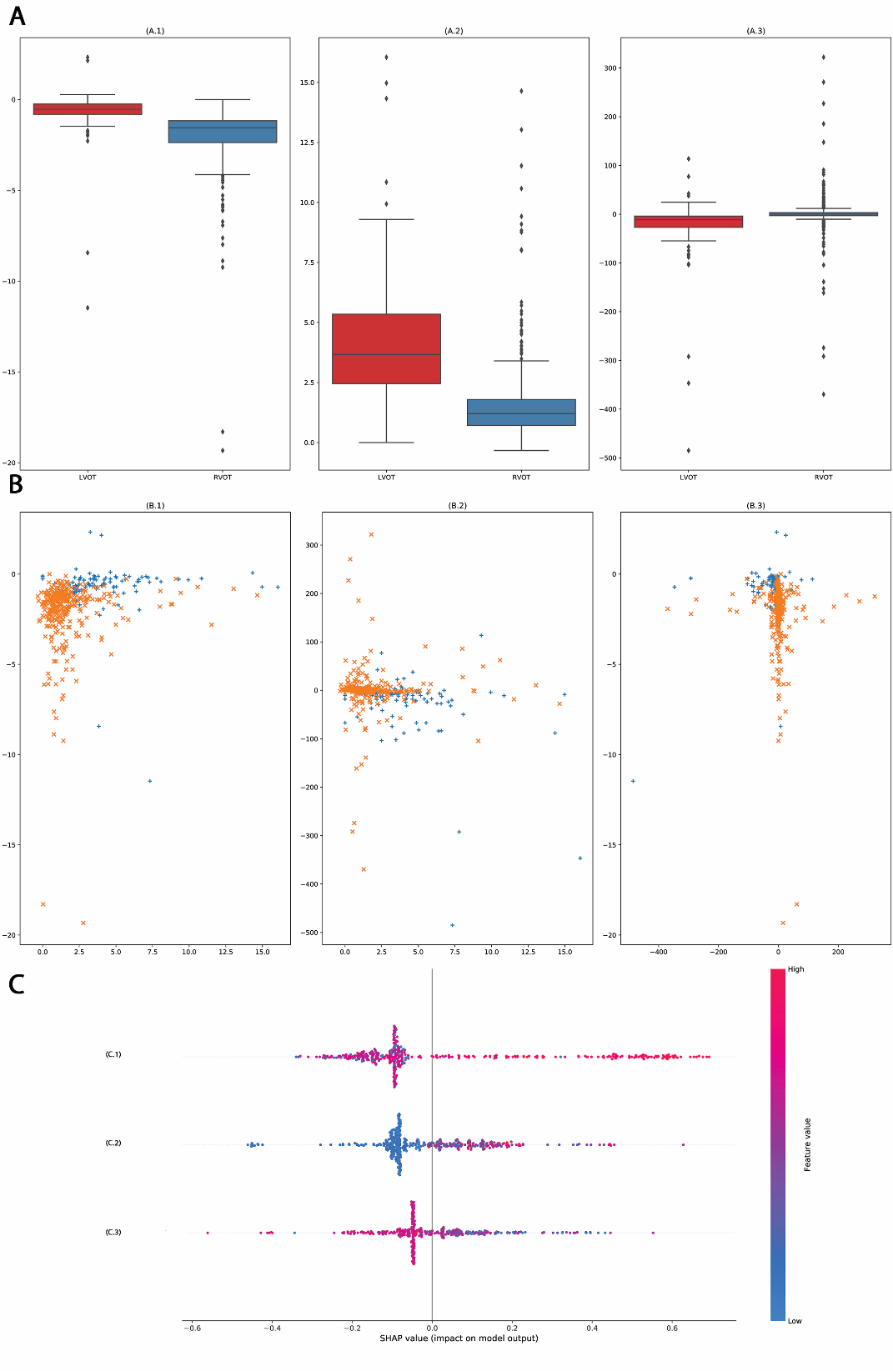


1. LCC = left coronary cusp; RCC = right coronary cusp; NCC =non-coronary cusp; AMC = aortomitral continuity; AC = anterior cusp; LC = left cusp; RC = right cusp; MV= mitral valve; TV = tricuspid valve. [↑](#footnote-ref-1)
2. SR = sinus rhythm; other abbreviations as in Table 1. [↑](#footnote-ref-2)
3. The abbreviations are as in Table 1. [↑](#footnote-ref-3)
